# Performance of Malaria Volunteers regarding Malaria Control Activities in Southeastern Myanmar: A Study in the Areas under Coverage of an Ethnic Health Organization

**DOI:** 10.1155/2021/6642260

**Published:** 2021-01-15

**Authors:** Ye Thiha, Thant Zin, Kyawt Mon Win, Myat Thu Soe, Pyae Linn Aung

**Affiliations:** ^1^STI Myanmar University Campus, University of Bedfordshire (UK), Yangon, Myanmar; ^2^Department of Public Health, Ministry of Health and Sports, Naypyitaw, Myanmar; ^3^Myanmar Health Network Organization, Yangon, Myanmar

## Abstract

Malaria volunteers (MVs) play an essential role in resolving malaria problems by delivering greater access to diagnosis and treatment services, mainly for the underserved community residing in hard-to-reach rural areas. The Karen Department of Health and Welfare (KDHW) has implemented community-based malaria control activities among the ethnic minorities in southeastern Myanmar by promoting the roles of MVs. This study aimed to explore the factors influencing the performance of MVs regarding malaria control activities in the area. From July to August 2019, a cross-sectional study was conducted in 12 townships of southeastern Myanmar under the umbrella of the KDHW malaria project. A total of 140 MVs were employed as study participants. Data were collected through face-to-face interviews using a structured questionnaire. For data analyses, descriptive statistics, chi-squared tests, and logistics regression models were applied. More than half of the MVs perceived a good level of performance on malaria control activities. A higher level of performance has been observed among the MVs who had another job (AOR: 1.9, 95% CI: 1.2–3.9), those experienced in health-related fields (AOR: 1.9, 95% CI: 1.4–4.9), who received good community support (AOR: 2.1, 95% CI: 1.3–10.9), who were volunteers beyond three years (AOR: 4.0, 95% CI: 2.8–9.2), and whose family income totaled over 500,000 MMK (AOR: 2.8, 95% CI: 1.6–4.2). The results mentioned the characteristics which should be prioritized in recruiting MVs. MV network and their workforce need to be nurtured by encouraging community support. For performance sustainability, attractive incentive schemes or a salary should be subsidized in support of their livelihoods.

## 1. Background

Malaria remains one of the public health problems in many countries, although being a curable and preventable disease. The World Malaria Report estimated 228 million malaria cases and 405,000 malaria deaths occurred globally during 2018 [1]. In Myanmar, malaria remained endemic in 291 out of 330 townships, and more than 60% of the total population were at risk of malaria infection [2]. Although there was remarkable progress in reducing malaria morbidity and mortality over the last decade, about 75,000 malaria cases and 19 malaria-related deaths were reported in 2018. This situation represented Myanmar as one of the most malaria endemic countries in the Greater Mekong Subregion [1]. Most of the reported malaria cases emerged from areas that were hard to reach in nature, occupied with poor transportation infrastructures, internal armed conflicts, or at cross-border points [3]. Health status in these locations appears to be struggling to improve and the outputs of implemented disease control services were very minimal.

Nevertheless, Myanmar has set a goal of being malaria transmission-free by 2030, with the encouragement of the World Health Organization (WHO) and the country's government [3, 4]. Thus, the National Malaria Control Program (NMCP) has set specific policies in the National Malaria Elimination Plan 2016–2030 [3], including achieving equity in access to services irrespective of gender, race, and ethnicity, especially for the most vulnerable and hard-to-reach populations. As part of this, the NMCP has implemented extensive malaria case surveillance systems at the village level by supporting the village volunteers' workforce. Currently, NMCP has trained and supported more than 15,000 active malaria volunteers (MVs) over the country in collaboration with nongovernment organizations, international partners, and Ethnic Health Organizations (EHOs) [2, 5]. A malaria volunteer refers to a person residing in hard-to-reach areas where no government health facilities exist and is performing health services voluntarily to the local community without pay [6]. In 2016, those MVs performed diagnosis and treatment for 73,404 malaria cases, accounting for nearly 70% of the country's achievement in community case management [2, 6]. That figure means the participation of MVs in Myanmar has been playing a major role in ensuring universal access to malaria diagnosis and treatment among hard-to-reach and at-risk populations (including mobile and migrants populations, forest-related workers usually residing in remote, cross-border, and ethnic groups), especially in rural areas.

The WHO has highlighted that early diagnosis and treatment serves as the most effective way to prevent an uncomplicated malaria case from developing into more severe conditions or death [7, 8]. This strategy was also important for eradicating the gametocide stage in the case of *Plasmodium falciparum* infection to interrupt possible onward transmission [7]. In an area with massive malaria caseloads, increased access to diagnosis and treatment facilities through MV posts would be an effective solution to reduce the disease burden within a reasonable time frame [9]. A systematic literature review also pointed out the importance of community health volunteers in implementing effective community case management for malaria [10]. As such, ensuring MVs' performance in optimum and regular needs assessment is important to conduct day-to-day malaria prevention and control activities smoothly and to reach country-wide universal health coverage. A study concluded that strengthening of knowledge and attitudes, the presence of family support, and delivering other motivational incentives acted as necessary components to create better roles of MVs in malaria control activities [11].

In an area with ongoing political instability and conflicts, malaria control services were suggested to be impossible to deliver through the government sector. For an intervention or activity, it might be critical to enter into a local community unless the involvement of local people from a particular location already existed [12]. For this reason, EHOs were found in the 1900s for the sake of health for ethnic minorities residing in conflict-affected areas which were mostly peripheral to the government health care services [13]. The EHOs trained and supported village MVs chosen from the local people. The organizations also distributed diagnostic tools and antimalarial drugs for MVs to serve as the frontline malaria service providers. Thus the capacity of EHOs should be properly strengthened for those isolated areas to sustain the progress towards the national malaria elimination target. Additionally, health care promotions in these postconflict areas would also strengthen the national reconciliation process. Therefore, this study aimed to evaluate the performance of MVs and its influences among the volunteers who provide malaria control activities among Kayin ethnic people under the coverage of an EHO in Myanmar.

## 2. Methods

### 2.1. Study Area

**Figure 1:**
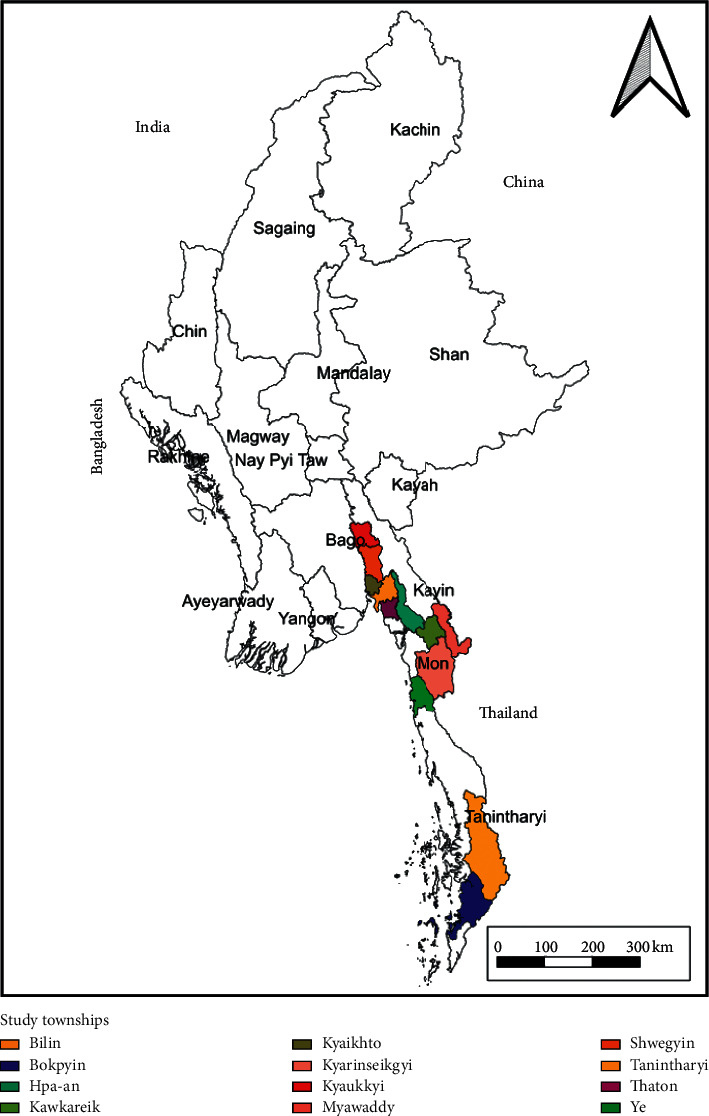
Study townships.

The study was purposely conducted in 12 townships of Southeastern Myanmar where the Regional Artemisinin Resistance Initiative to Elimination (RAI-2E) malaria project through the Global Fund has been implemented by the Karen Department of Health and Welfare (KDHW), one of the EHOs in Myanmar. These 12 townships included Shwegyin, Kyaukkyi, Hpa-an, Kawkareik, Myawaddy, Kyarinseikgyi, Thaton, Billin, Kyaikhto, Ye, Tanintharyi, and Bokpyin (Figure 1). All these townships were recorded as hard-to-reach areas according to road accessibility across seasons assessed by the General Administration Department [14]. These locations were selected to conduct the research due to the unique geographical nature of the areas and the presence of similar population characteristics. The townships were known for being malaria endemic, remote, and where the capacity of the health care providers including the MVs regarding malaria control activities had not yet been analyzed. Furthermore, the area served as a malaria hot spot due to the enormous cross-migration of the population being the border areas of Thailand and Myanmar.

The KDHW had trained and supported an average of 25 volunteers in each township, totaling 290 volunteers, to provide malaria diagnosis and treatment among hard-to-reach and at-risk populations especially Kayin ethnic people of rural communities from 12 townships of southeastern Myanmar. Generally, four criteria to select the villages were usually used to deploy a volunteer [5]: (i) village with high malaria burden, (ii) hard-to-reach location, (iii) village with relatively high population density, and (iv) village where no health care facilities or health staff close by. Next, to select a MV [5, 6], the preferred individuals should be (i) a permanent resident of a village, (ii) accepted by the community, (iii) willing to work as volunteers, (iv) male or female with 18 years to 50 years of age, and (v) able to read and write Myanmar language and speak local dialects. In addition, recommendations from respective village administrative groups were also considered while recruiting the volunteers. Moreover, overlapping with other similar projects was avoided.

After choosing a volunteer, the organization provided a five-day recruitment training for malaria case management and reporting mechanisms. After training, the volunteers were allowed to use rapid diagnostic test (RDT) (SD BIOLINE Malaria Ag *P*. *f*/*P*. *v*) kits and to treat uncomplicated malaria cases according to the national malaria treatment guidelines. Volunteers were encouraged to refer the cases (complicated malaria, malaria with pregnancy, or <1-yr-old children). The key activities of volunteers included but were not limited to (i) providing early diagnosis and treatment of uncomplicated malaria cases, (ii) referring severe and complicated malaria patients to the nearest hospital or health facilities, (iii) distributing materials like pamphlets, posters, and long-lasting insecticide-treated nets (LLINs) to community people, (iv) educating people to promote malaria prevention and control, (v) observing proper LLIN use by the community, and (vi) recording and reporting of patients credentials. Routine on-the-job training as well as annual refresher courses were also organized.

The KDHW supported the incentives, either monetary (∼15USD monthly) or nonmonetary rewards such as umbrellas, backpacks, hats, and torch lights, for the volunteers depending on the instruction of the national malaria control program or budget availability. The organization also delivered monthly supportive monitoring and evaluation visits by field or central supervisors to ensure the quality of malaria care services as well as data recording and reporting and to solve any issues or challenges faced by the assigned volunteers.

### 2.2. Sampling Procedures

The 290 volunteers, who had completed the training and were providing malaria health care services to the community in the 12 selected townships under the supervision of the KDHW for at least six months, were eligible to participate in this study. However, those refusing to participate in the interview and those unable to communicate well due to serious illness were excluded from the study. The sample size was determined by using Epi Info™-7 Software by putting the proportion of volunteers who revealed the good performances in Myanmar at 44.0%, as stated in one study [11]. Then the required sample size should be at least 140 by adding 10% more for the dropout and nonrespondent rate. A list of eligible volunteers separated by township was requested from the KDHW. To achieve the desired sample size, an average of 12 volunteers from each township was selected using systematic random sampling through a number lottery method.

### 2.3. Study Tools

The study deployed primary data collection through a quantitative cross-sectional design using a structured questionnaire. The questionnaire was adapted from previous relevant studies [11, 15] and another tool [16]. It was firstly developed in English and then translated to Myanmar language by the research team. The questionnaire was divided into six parts described below.

  Part I: sociodemographic factors of the respondents—the nine questions included age in years, sex either male or female, marital status, educational level, types of occupation, average annual family income, related working experience, and duration as volunteer. The answers were predefined as ministatements and listed under each question.
  Part II: knowledge—this part consisted of 13 miniquestions regarding procedures of RDT testing, treatment guidelines, and information on LLINs. The questions consisted of both positive and negative statements. The answers could be “yes,” “no,” or “do not know.” For the correct answer, the respondents received “1” score but received “0” score if they gave an incorrect answer or responded as “do not know.”
  Part III: perception of roles and functions of volunteer—the six questions included both positive and negative structures. By using a three-point Likert's rating scale [17], the scores were given in the order of “3,” “2,” and “1” for “agree,” “uncertain,” and “disagree,” respectively, for positive statements. Reciprocally, the scores of “1,” “2,” and “3” were assigned for “agree,” “uncertain,” and “disagree” in case of negative statements.
  Part IV: family support—this section included four questions. Only one choice was allowed, and the scoring criteria were always = 3, sometimes = 2, and never = 1 for positive questions or always = 1, sometimes = 2, and never = 3 for negative questions.
  Part V: community support—this part was constructed using four questions and the answers included “always,” “sometimes,” and “never” according to the three-point Likert's scale model. The scores were formulated as “3” for “always,” “2” for “sometimes,” and “1” for “never,” accordingly.
  Part VI: performance of the volunteers—this was defined as the volunteers insights upon their current practices regarding malaria control activities guided by the organization. The activities were firstly constructed based on the village volunteer algorithm endorsed by the national program [6]. Some activities were narrowed down and skipped upon considering several relevant studies and reports [6, 11, 15, 16, 18] and also their reliability in the actual field situation. Finally, 11 activities represented as the key activities of a MV which overall addressed performing blood testing, prescribing of antimalarial medicines, operating health education sessions, delivering preventive measures, and the last for data recording and reporting data. The answers were structured as a three-point Likert's scale using “always,” “sometimes,” and “never.” The scoring system (always = 3, sometimes = 2, never = 1) was applied.

The overall scores of the level of knowledge level, perception level, family and community support, and performance were compiled and categorized into two groups using Bloom's Taxonomy theory [19] as <80 of total score = poor level and ≥80% of total score = good level.

### 2.4. Data Collection

Two research assistants were hired among the local residents who attained at least a bachelor's degree and fluency in local language and tones. They were trained for two days about the nature of the study including the flow of the questionnaire, data collection process, and research ethics in conducting study involving human subjects. Practical sessions were also demonstrated to reflect the real situation and to be aware of the interviewing time. In this study, data were collected at the township level during the quarterly coordination meetings conducted by KDHW during July and August 2019. In the early morning before meeting time, each selected volunteer's name was announced and requested to receive an interview. Two research assistants conducted face-to-face interviews with the validated questionnaires in a private room away from other people. The interviewing environment was also secured from other people entering to avoid any annoyance. An interview normally took not more than 30 minutes.

### 2.5. Data Processing and Analysis

The consistency, completeness, and integrity of the data were checked immediately by the researchers after collecting data daily. Data were encoded and entered into EpiData software. After that, all the data were transferred and analyzed using Statistical Package for the Social Sciences (SPSS, version 23). Frequencies, proportions, means, and standard deviations for descriptive analysis were used to describe sociodemographic factors, knowledge, perception of volunteers regarding malaria, family and community support, and the performance of volunteers. To determine significant differences between dependent and independent variables, bivariate analysis using chi-squared tests were applied. To obtain absolute chances for study variables and avoid including too many redundant variables, the variables which showed *p* value <0.05 during chi-squared tests were analyzed in simple and multiple logistics regression models to demonstrate the odd ratios and 95% confidence intervals.

## 3. Results

### 3.1. Sociodemographic Characteristics of the MVs

This study deployed a total of 140 malaria volunteers. The ages of the volunteers ranged from 18 to 50 years. The majority (71.5%) was under 30 years. About two-thirds were married female. Most participants attained secondary school and lower level education. For occupation, 32.1% worked in an agricultural related occupation followed by owning a business (28.6%) and the daily wages laborers (20.0%). Many (62.1%) earned more than 500,000 MMK as annual family income ranging from 150,000 MMK as minimum and 1,800,000 MMK as maximum. Most respondents (65.7%) did not posses no working experience of health-related work before being a MV. Regarding the time of being a volunteer, more than 81% of respondents worked as a volunteer for more than one year ranging from one to six years (Table 1).

### 3.2. Knowledge about Malaria Control Activities

Regarding knowledge on current using the malaria RDT, almost all the study's respondents correctly answered about the storage of malaria RDT at the coolest place where the temperature was less than 40°C, including the drops of buffer solution to be placed in the RDT. However, only 75.0 and 85.7% of malaria volunteers gave the right answers on the time frame for reading results and concluding the results based on the bends appearing in the RDT, respectively. For questions concerning prescription antimalarial drugs, all respondents knew well about referring severe or complicated malaria cases to the nearest hospitals or health care facility. More than 90% of volunteers delivered the correct dosage schedule to treat *P. falciparum*, *P. vivax* and mixed infections accordingly. However, some often prescribed antimalarial medicines among patients with negative RDT results. Moreover, more than 99% of respondents knew that using LLINs was the most effective way to prevent mosquito bites and that sleeping under LLIN was the best way to prevent malaria. Next, more than 92% were aware that the effectiveness of LLINs exceeded beyond one year. Interestingly, more than 70% believed that LLINs could cause skin itchiness and respiratory tract infection, particularly among children (Table 2).

### 3.3. Perceptions towards Roles and Functions of the MVs

Among the statements addressing the perceptions of the studies volunteers towards their roles and functions, all respondents agreed with the importance of timely referral for severe or complicated malaria cases to receive life saving measures, building good communication mechanisms within community was crucial and the last, they served essential roles in delivering malaria control activities and preventing the emergence of drug resistant malaria. Further, 13.6% of the respondents did not realize that the disseminating of health knowledge was a necessary tool for malaria prevention and control. Next, 29.3% disagreed that timely submission of records and reports was important. Last, 22.9% thought that the assessment on the proper use of LLINs in the community was unnecessary (Table 3).

### 3.4. Family and Community Support for the MVs

As shown in Table 4, most respondents (82.1%) always received kind encouragements from their family members to work as a MV. However, 45.7% of respondents revealed their family members sometimes accompanied them in implementing activities. About 93% of the respondents never received complaints from family members on decreased time spent with the family and lower family income because of the volunteer service. Regarding community supports, 80% of the respondents received encouragement from the villagers to work as a malaria volunteer. Around two-thirds of the participants also described the community support regarding the aspects of providing information on new suspected malaria cases, active participation in malaria control activities, and following treatment procedures and preventive methods.

### 3.5. Performance of MVs regarding Malaria Control Activities

Performance of MVs regarding malaria diagnosis was assessed. Most respondents (82.1%) performed RDT testing for every malaria-suspected case. When handling test kits, 93.6% followed the procedures. The volunteers also strictly followed the malaria treatment guidelines. Most (98.6%) provided the instructions for how to take the medicines as well. More than 99% usually referred to every severe or complicated malaria cases to higher level facilities. Despite 89.3% admitting that they delivered the health messages while waiting for the RDT result for 15 minutes, some (23.6%) did not distribute the education aids materials like pamphlets and posters. More than 87% alluded that they were always involved in LLINs distribution. Almost all the volunteers (98.6%) also carefully recorded the required information in the detailed Case Report Forms (CRFs). However, many participants (30.7%) had never observed the LLIN use by the community. All the respondents submitted the records and reports according to the targeted time frame, either by month or by quarter (Table 5).

### 3.6. Overall Levels of Knowledge, Attitude, Family Support, Community Support, and Performance of MVs

Table 6 summarizes the overall levels of knowledge, attitude, family support, community support, and performance of the studied MVs. Two groups for each variable were constructed. Most respondents (85.7%) showed a good level of knowledge; similarly, 87.1% expressed positive perception. Regarding family and community support, 72.1% of respondents received good family support, while 67.1% maintained good support from the respective community. Additionally, 53.6% of the respondents perceived good level of performance.

### 3.7. Relationships between Sociodemographic Factors, Knowledge, Attitude, Family Support, Community Support, and Performance of MVs

The cross tabulations were conducted between the performance of MVs and other variables including sociodemographic characteristics of the respondents, their levels of knowledge, attitude, and receiving support from family and community. The higher the age above 30 years, the greater proportions of good level performance were observed. Female volunteers revealed higher possession of good roles than those of male respondents. Similarly, married MVs who attained above primary level education and possessed a job in addition to a volunteer work and family income of >500,000 MMK had better performance. Next, those who had previous experience of working in a health related area before being a volunteer and volunteers who worked in this field for more than one year were likely to show better performance. Last, MVs with a background of good community support demonstrated good performance roles (Table 7).

The possible relationships were addressed by the chi-squared test as shown in Table 7. These results were insignificant concerning some sociodemographic characteristics of the respondents such as age, sex, marital status, level of education, level of knowledge, level of attitude and family support, and performance of MVs. Further, statistically significant differences were observed among types of occupation in addition to volunteer work (*p*=0.002), ranges of annual family income (*p*=0.010), time of being a volunteer (*p*=0.034), possessing working experience related to health work before serving as a volunteer (*p*=0.025), and performance levels regarding malaria control activities at *p*-value <0.05.

### 3.8. Logistic Regression Models

Both simple and logistic regression models were applied. In regression analysis, the performance of MVs showed approximately twofold increments among employed MVs in addition to volunteer position (AOR: 1.9, 95% CI: 1.2–3.9), individuals experienced in a health related field before serving as a volunteer (AOR: 1.9, 95% CI: 1.4–4.9), and those who received good support from their communities (AOR: 2.1, 95% CI: 1.3–10.9). Moreover, a longer time being a volunteer served as a backbone for better performance, including MWs with 1 to 3 years' experience (AOR: 1.9, 95%CI: 1.5–2.9) and MWs with more than 3 years' experience (AOR: 4.0, 95%CI: 2.8–9.2). Furthermore, the greater odds were also observed among these respondents whose family income totaled more than 500,000 MMK, towards good levels of performance (AOR: 2.8, 95% CI: 1.6–4.2) (Table 8).

## 4. Discussion

This study investigated the performance of MVs who devoted their lives as frontline malaria service providers especially in rural and remote areas of Myanmar where government health care services were scarcest. The results summarized that more than one-half of the studied MVs provided a good level of performance in implementing malaria control activities. The factors enhancing the roles of the MVs were being employed rather than solely providing volunteer work, having experience in a health related sector before serving as a volunteer, a longer time serving as a volunteer, and having family income totaling over one half million MMK. Nevertheless, other variables such as age, sex, educational attainments, marital status, knowledge levels, attitude levels, and family support suggested only insignificant relationships with MV performance. The majority population in Myanmar lives below the poverty line and generally cannot access quality health care and encounters high out-of-pocket expenditures [20]. On the contrary, the current health system in Myanmar has been considered fragmented and unstable at the system level [21]. In addition, universal health coverage remains fragile [20]. These continue to threaten the achievement of sustainable development goals for the country. Meanwhile, MVs serve as the first contact point for delivering health services to vulnerable populations by increasing access to diagnosis and treatment facilities. According to one country report, around 70.0% of patients with malaria were tested and treated by the volunteer workforce [2]. In an area with high malaria caseloads, the burden has been progressively declined after introducing the MVs [9]. Using an integrated approach, the volunteers performed very well for symptomatic treatment of other minor illnesses like seasonal flu and referrals of major infectious diseases like *tuberculosis* and HIV [22]. However, the roles of volunteers should be sustained once malaria problems decreased, mainly during the final stages of the malaria elimination era. Otherwise, malaria elimination might not be attained by the targeted timeline of 2030. Therefore, understanding the factors which might influence the performance of the volunteers would have a certain advantage in strengthening the roles of volunteers in the upcoming elimination trajectory.

MVs were mainly responsible for routine case surveillance using the passive case detection approach, i.e., when a patient with malaria-like symptoms sought treatment at the volunteer's post, they provided a blood test and prescribed treatment as necessary. Malaria is highly seasonal and the trend was usually high during rainy seasons (July-October) [23]. Therefore, before or after malaria peak seasons, the volunteers generally had less workload and did not need to contribute full-time volunteer work unless their respective organizations had assigned other duties. A volunteer could easily spare some time to work in another job especially to increase family income. It might also reduce the production of complaints by other family members concerning lower income or less family bonding time because of volunteer work. Other studies have confirmed a similar finding that volunteers with another source of income or a paid job tended to show good performance [11, 24]. In a populated village or an area with very high malaria risks, the recruitment of more than one volunteer would be more efficient. Instead of organizing frequent township level meetings or training, on-site training during monitoring visits was recommended. However, a well-designed backup plan should be in place to substitute the low performance volunteers who were busy with nonvolunteer work and failed in routine reporting or showed zero reporting for some consecutive months without strong justification.

MVs voluntarily performed malaria-related health services in the community without pay. However, the organization routinely supported monthly incentives in line with the guidelines from the national program. Yet, the MVs were also satisfied with the current amount of payment. Nevertheless, the result of this study suggested that the volunteers who retained higher family income were likely to exhibit greater performance levels. Of course, a person working as a volunteer without pay might experience stress for the well-being of other members. Once family members have established a backup for gross family income, the volunteer could focus on malaria control activities for the sake of improving community health. A study in Myanmar also formulated the unique fact that volunteers with high family income delivered good roles in implementing malaria control activities [11]. Because malaria programs are vertical in nature, depending on under which auspice they are in, the level of support and type of support they received from their organizations might differ according to the donor's policies, budget allowance, and organizational rules. The national program should endorse a standardized rate that can entirely cover the efforts made by the volunteers. Whenever possible, this rate should reflect the minimal daily wage of a citizen (i.e., 7,000 MMK/day), approved by the Ministry of Labor [25]. Alternatively, when selecting volunteers, their family income should be informally assessed.

The MVs having health related working experience showed better roles than those volunteers without health related working experience. The finding was similar to that of another study conducting a country-wide survey in Myanmar [11]. Local people certainly realized a person who was very active in the community worked with underlying better health knowledge than other villagers. Therefore, they usually pointed out that person to help or be involved in health related activities organized by various organizations. Subsequently, 34.3% of studied participants possessed health related working experience. Those individuals were aware of the nature of health interventions and demonstrated good communication skills regarding health care projects and organizations. They might receive job-related training as well. These experiences might enhance the volunteers in absorbing the knowledge transferred during the training and also helped them to easily follow the diagnostic procedures [26]. Individuals possessing health related experience should be given precedence in being selected as a volunteer. However, recruiting a volunteer by many organizations should be precluded to avoid works overload, data repetition, and time conflicts using a different scope of assigned work.

Generally, a person who decided to commit to a single job for a longer time would then gradually become more skillful than that an individual who frequently changed their positions at different work places. The underlying reasons included but were not limited to underlying enthusiastic mindsets, attending relevant training, receiving job-related experiences, and sharing knowledge among colleagues and mentorships from immediate supervisors. Likewise, in this study, the volunteers with more than one year's experience represented better roles in implementing malaria control activities. A similar result could be observed in another study in which volunteers with longer experience showed more appropriate roles in their performance [11]. The longer the time of being a volunteer, the more people recognized the performance of volunteers and appreciated the existence of MVs within the community in controlling and preventing deadly diseases [27]. The appreciation from the respective community would be another reason for MVs to perform good roles. To offset the low attrition rate among trained MVs, each organization might want to implement an award system for higher performing volunteers. Introducing an output-based incentive scheme under proper supervision might also be a solution. In the case volunteers moved to another location though they still wanted to participate as a volunteer, their names should be referred to the organization that operates in the new location.

Whenever village-based health interventions were delivered, the MVs were critical to operating activities smoothly. Given that fewer human resources were available in the health sector, support from the villagers was usually encouraged to accelerate the activities. Similarly, the work of volunteers could not succeed without proper cooperation from the community, mainly for adhering to treatment procedures, following preventive measures especially the use of bed nets, seeking diagnostic testing in time, and attending health education sessions [28]. In this study, the volunteers with good community support performed better than those receiving poor support. The finding was supported by a study conducted in Myanmar and Malawi [29, 30]. Once the volunteers received the respect of the community, they would strengthen future activities. The respective organization should advocate the community members and village stakeholders about the roles of volunteers in the villages and offer support by explaining the importance of community involvement in malaria control activities delivered by the MVs. Additionally, some activities like organizing awareness-raising campaigns, LLINs distribution, and surveying its use should be accompanied by higher level officials together with the MVs.

Because this constituted a cross-sectional quantitative study, like any survey, any causal relationships between influencing factors and the performance status of malaria volunteers were unable to confirm. A qualitative interview may be warranted to understand the contextual factors behind their performance. As the responses were self-reported, to minimize any bias, it would be better to introduce other validation steps in accessing their roles, for example, validate their past records, treatment compliance, reporting regularity, testing performances, and other performance benchmarks, and then triangulate with their knowledge, skills, and perceptions. This study only assessed MVs participating under the KDHW. Thus, the ability to generalize the findings would be limited as this study could not provide any findings from different organizations that delivered different award systems, program designs, monitoring and reporting systems, incentives, and supervisors although each organization might implement uniform treatment guidelines, diagnosis, and referral system. The finding was also subject to bias in particular concerning assessing the community members' participation and family members' support which were subjective and comprised just the opinions of the volunteers. The volunteers might be reluctant to openly disclose about collaborating with village leaders and the armed groups due to fear of dismissal, as such findings on high community participation level might have been biased. Due to the quantitative nature of the study, providing an in-depth understanding of the problem is impossible.

## 5. Conclusion

The study summarized those characteristics which should be prioritized in future recruitments of MVs. MVs networks and their workforce need to be nurtured by encouraging community support. To eliminate malaria, indisputably, these VHV networks and workforce need to be strengthened as only these mechanism exist to ensure universal coverage of malaria services in all underserved communities across the country. Current MVs are unengaged as employees recognized by any health system (either government or NGO), but rather are supported as volunteers and only minor compensation was made as recognition of the time they devoted to delivering health services. Regarding performance sustainability, attractive incentive schemes or a salary should be subsidized to support their livelihoods. However, the current incentives were still perceived as good motivational factors for the MVs, so that the national program needs to review the appropriateness of such schemes while balancing the economy, value for money, and sustainability. Last, “health constitutes a bridge for peace,” supporting the health of the community in nongovernment controlled areas representing the door to further continue the ongoing peace building process in Myanmar.

## Figures and Tables

**Table 1:** Sociodemographic characteristics of the 140 respondents.

Characteristics	*n*	%
*Age (years)*		
18–30	100	71.5
31–40	24	17.1
>41	16	11.4
Mean ± SD	28.2 ± 8.9				
Min–Max	18–50				

*Sex*		
Male	57	40.7
Female	83	59.3

*Marital status*		
Single	53	37.9
Married	87	62.1

*Education*		
Primary school	44	31.4
Secondary school	48	34.3
High school	42	30.0
College/Bachelor	1	0.7
Others (monastery education/illiterate)	5	3.6

*Major occupation (apart from malaria volunteer work)*		
Unemployed	6	4.3
Agriculture/farmers	45	32.1
Own business	40	28.6
Dependent/housewives	21	15.0
Daily wages labors	28	20.0

*Annual family income (MMK)* ^*∗*^		
≤500,000	53	37.9
>500,000	87	62.1
Mean ± SD	87,0071 ± 95,0084
Min–Max	150,000–1,800,000

*Working experience related to health before being a malaria volunteer*		
Ever	48	34.3
Never	92	65.7

*Time of being a malaria volunteer (year)*		
≤1	25	17.9
1–3	69	49.3
>3	46	32.8
Mean ± S.D	1.80 ± 0.401	
Min–Max	0.5–6	

^*∗*^1,300 MMK ∼ 1 USD, SD: standard deviation, Min: minimum, and Max: maximum.

**Table 2:** Correct knowledge about malaria control activities (*n* = 140).

Knowledge statements	Correct answer	
	*n*	%
*Malaria rapid diagnostic test (RDT)*		
Keep RDTs in cool place of your residence (within 1–40^o^C)	135	96.4
^*∗*^Put 8 drops of buffer solution into the buffer well perpendicularly	140	100
Read RDT's results within 15–30 minutes	105	75.0
If RDT appeared lines at C&*P. f*, the result is *P. falciparum*	120	85.7
*Prescribing antimalarial medicines*		
*P. falciparum*-artemisinin combination therapy (ACT) (3 days) + primaquine (stat on first day)	133	95.0
*P. vivax*-chloroquine (3 days) + primaquine (weekly for 8 consecutive weeks)	134	95.7
Mixed infection-ACT (3 days) + primaquine (weekly for 8 consecutive weeks)	131	93.6
^*∗*^RDT (−)ve patients-antimalarial medicine should be prescribed	136	97.1
Severe or complicated malaria-referral	140	100
*Long lasting insecticide treated nets (LLINs)*		
Using LLINs is the most effective way to prevent mosquito bites.	139	99.3
Sleeping under LLIN is the best way to prevent malaria.	139	99.3
^*∗*^Sleeping under LLIN can cause skin itchiness and respiratory tract infection among children	38	27.1
^*∗*^LLIN can be effective for one year only	129	92.1

^*∗*^Negative questions; RDT: rapid diagnostic test.

**Table 3:** Perceptions towards roles and functions of malaria volunteers (*n* = 140).

Statements	Agree		Disagree		Uncertain	
	*n*	%	*n*	%	*N*	%
Giving knowledge on malaria to local people individually and in a group is a necessary tool for the prevention and control of malaria	121	86.4	19	13.6	0	0

Timely submission of patients records and reports is important	99	70.7	41	29.3	0	0

Timely referral for severe malaria cases is essential for life saving	140	100	0	0	0	0

It is necessary to build good communication with community	140	100	0	0	0	0

Malaria volunteers play an important role in implementing malaria control activities and prevention of drug resistant malaria	140	100	0	0	0	0

^*∗*^Monitoring the proper use of LLINs in the community should not be done	32	22.9	104	74.3	4	2.8

^*∗*^Negative statements.

**Table 4:** Family support and community support for the malaria volunteer (*n* = 140).

Descriptions
	Always		Sometimes		Never	
	*n*	%	*n*	%	*n*	%
*Family support*						
Members of your family encourage you to voluntarily work as a malaria volunteer	115	82.1	23	16.4	2	1.5
Members of your family join you in your activities	61	43.6	64	45.7	15	10.7
^*∗*^Working as a volunteer, your family complains of lesser family income	10	7.1	0	0	130	92.9
^*∗*^Working as a volunteer, your family complains about decreased time spent on them	8	5.7	2	1.4	130	92.9

*Community support*						
People in your village encourage you to voluntarily work as a malaria volunteer	112	80.0	19	13.6	9	6.4
People in your village informed you for the new suspected cases of malaria	96	68.6	35	25.0	9	6.4
People in your village actively participated in all of your activities	87	62.2	45	32.1	8	5.7
People in your village adhered on malaria treatment, prevention, and control guidelines	95	67.9	41	29.3	4	2.8

^*∗*^Negative statements.

**Table 5:** Performance of 140 respondents regarding malaria control activities.

Activities	Always		Sometimes		Never	
	*N*	(%)	*N*	(%)	*N*	(%)
*Malaria diagnosis*						
Diagnose every suspected cases of malaria with RDT	115	82.1	25	17.9	0	0
Strictly follow the procedure of RDT testing	131	93.6	9	6.4	0	0
*Malaria treatment*						
Follow the guidelines of the malaria treatment	140	100	0	0	0	0
Give instruction on full course of malaria drugs	138	98.6	2	1.4	0	0
Refer every severe malaria case to the nearest hospitals/health facilities	139	99.3	1	0.7	0	0
*Health education*						
Provide education on malaria control and prevention individually while waiting for RTD's result	125	89.3	15	10.7	0	0
Distribute pamphlets and posters to the RTD tested patients as well as community	80	57.1	33	23.6	27	19.3
*Malaria prevention*						
Properly distribute LLINs to the community depend on the availability of resources	122	87.1	16	11.4	2	1.5
Observe how people use LLINs effectively	43	30.7	54	38.6	43	30.7
Record patients characteristics, RDT result and prescribed medications correctly in CRFs	138	98.6	2	1.4	0	0
*Data recording and reporting*						
Submit records and reports as scheduled	139	99.3	1	0.7	0	0

RDT: rapid diagnostic test, LLIN: long-lasting insecticide-treated net, CRF: case report form.

**Table 6:** Level of knowledge, level of attitude, family support, community support, and performance of malaria volunteers (*n* = 140).

Descriptions	*n*	%
*Levels of knowledge*		
Good	120	85.7

Need to improve	20	14.3

*Levels of perception*		
Positive perception	122	87.1

Negative perception	18	12.9

*Family support*		
Good	101	72.1

Poor	39	27.9

*Community support*		
Good	94	67.1

Poor	46	32.9

*Performance of the malaria volunteer*		
Good	75	53.6

Poor	65	46.4

Grouping by Bloom's Taxonomy theory, good level: ≥80% of the total score; poor level: <80% of the total score.

**Table 7:** Relationships between sociodemographic factors, knowledge, attitude, family support, community support, and performance level of malaria volunteer (*n* = 140).

Descriptions	Level of performance				*p* value
	Good		Poor		
	*n*	%	*n*	%	
*Age (years)*					
18–30	52	52.0	48	48.0	0.56
31–40	13	54.2	11	45.8	
>41	10	62.5	6	37.5	
*Gender*					
Male	27	47.4	30	52.6	0.22
Female	48	57.8	35	42.2	
*Marital status*					
Single	25	47.2	28	52.8	0.23
Married	50	57.5	37	42.5	
*Education*					
Primary and below	23	46.9	26	53.1	0.24
Above primary	52	57.1	39	42.9	
*Major occupation (apart from volunteer work)*					
Unemployed	4	66.7	2	33.3	0.002 ^*∗*^
With job	71	53.0	63	47.0	
*Annual family income (MMK)* ^*∗∗*^					
≤500,000	21	39.6	32	60.4	0.010 ^*∗*^
>50,0000	54	62.1	33	37.9	
*Working experience related to health before being as a volunteer*					
Ever	32	66.7	16	33.3	0.025 ^*∗*^
Never	43	46.7	49	53.3	
*Time period of being as a volunteer (year)*					
≤1	10	40.0	15	60.0	0.034 ^*∗*^
1–3	37	53.6	32	46.4	
>3	28	60.9	18	39.1	
*Knowledge level*					
Good	66	55.0	54	45.0	0.406
Need to improve	9	45.0	11	55.0	
*Attitude level*					
Positive perception	67	54.9	55	45.1	0.281
Negative perception	8	44.4	10	55.6	
*Family support*					
Good	59	58.4	42	41.6	0.064
Poor	16	41.0	23	59.0	
*Community support*					
Good	60	63.8	34	36.2	0.001 ^*∗*^
Poor	15	32.6	31	67.4	

^*∗∗*^1,300 MMK ∼ 1 USD; degree of freedom for chi-square test = 1;  ^*∗*^significance at *p* < 0.05.

**Table 8:** Influencing factors for the performance of malaria volunteers (*n* = 140).

Descriptions	COR	95% CI	AOR	95% CI
*Major occupation (apart from volunteer work)*				
Unemployed	1	—	1	—

With job	3.2	(2.1–4.2)	1.9	(1.2–3.9)

*Annual family income (MMK)* ^*∗*^				
≤500,000	1	—	1	—

>500,000	2.9	(2.0–4.6)	2.8	(1.6–4.2)

*Working experience related to health before being a malaria volunteer*				
Never	1	—	1	—

Ever	2.4	(1.8–4.5)	1.9	(1.5–2.9)

*Time of being a malaria volunteer (year)*				
≤1	1	—	1	—

1–3	2.6	(1.8–5.2)	1.9	(1.4–4.9)

>3	4.1	(3.1–9.2)	4.0	(2.8–9.2)

*Community support*				
Poor	1	—	1	—

Good	2.9	(1.4–11.2)	2.1	(1.3–10.9)

^*∗*^1,300 MMK ∼ 1 USD, COR: crude odds ratio by simple logistic regression, AOR: adjusted odds ratio by multiple logistic regression, and CI: confidence interval.

## Data Availability

All the data analyzed for this study are included within the article.

## Abbreviations

AOR: Adjusted odds ratio
CI: Confidence interval
COR: Crude odds ratio
CRF: Case report form
EHO: Ethnic Health Organization
KDHW: Karen Department of Health and Welfare
LLIN: Long-lasting insecticide-treated net
MVs: Malaria volunteers
NGO: Nongovernment organization
NMCP: National Malaria Control Program
RAI: Regional artemisinin resistance initiative
RDT: Rapid diagnostic test
SPSS: Statistical Package for the Social Sciences
WHO: World Health Organization.
